# The Dark Side of Emotion Recognition – Evidence From Cross-Cultural Research in Germany and China

**DOI:** 10.3389/fpsyg.2020.01132

**Published:** 2020-07-09

**Authors:** Helena S. Schmitt, Cornelia Sindermann, Mei Li, Yina Ma, Keith M. Kendrick, Benjamin Becker, Christian Montag

**Affiliations:** ^1^Department of Molecular Psychology, Institute of Psychology and Education, Ulm University, Ulm, Germany; ^2^Student Counseling Center, Beijing University of Civil Engineering and Architecture, Beijing, China; ^3^State Key Laboratory of Cognitive Neuroscience and Learning, IDG/McGovern Institute for Brain Research, Beijing Key Laboratory of Brain Imaging and Connectomics, Beijing Normal University, Beijing, China; ^4^The Clinical Hospital of Chengdu Brain Science Institute, MOE Key Laboratory for Neuroinformation, University of Electronic Science and Technology of China, Chengdu, China

**Keywords:** dark triad of personality, Emotional Manipulation, dark EI, reading the mind in the eyes test, emotion recognition, cross-cultural

## Abstract

**Background:**

The dark triad of personality (DT) comprises three antisocial personality traits (i.e., narcissism, Machiavellianism, and psychopathy) that are characterized by callousness and the motive to elevate the self while derogating other people. Previous research indicates that the positive relationship between the DT traits and interpersonally deviant behaviors is especially pronounced at high levels of emotional abilities. This has also been referred to as dark Emotional Intelligence (EI). Since prior studies predominantly examined dark EI via trait-approach, the present study targeted at providing evidence for dark EI using a behavioral measure of EI, namely emotion recognition performance. In order to study the robustness and cross-cultural validity of findings, parallel investigations were conducted in Germany and China.

**Methods:**

A total of *N* = 198 German (age: *M* = 23.40, *SD* = 5.88, 130 female) and *N* = 223 Chinese (age: *M* = 19.01, *SD* = 1.06, 105 female) participants took part in an online survey and completed a set of questionnaires in German and Mandarin translations, respectively. DT traits were assessed by means of the Short Dark Triad Scale. As a behavioral measure of emotional abilities, participants completed the Eyes Test for pairs of eyes of Caucasian and Asian models. Moreover, participants filled in the Emotional Manipulation Scale for the assessment of emotionally manipulative tactics.

**Results:**

Effects were highly gender- and culture-dependent. Among German females, Machiavellianism and narcissism showed the strongest positive associations with emotionally manipulative tactics at high levels of emotion recognition performance. A similar pattern of results was found among German males for psychopathy. None of the effects was observed in the Chinese female or male samples.

**Discussion:**

The present findings indicate that emotional abilities may constitute risk factors with the potential to promote rather than to prevent deviant behaviors especially in samples from Western cultures with pronounced scores on DT personality traits. Limitations and psychometric properties are discussed.

## Introduction

Dishonesty, interpersonal manipulation, betrayal, bullying and other forms of antisocial and unethical behaviors represent some of the greatest personal and societal challenges worldwide. While the media often report about the most sensational cases, a substantial proportion of the normal population violates moral rules and social norms every day (Shalvi et al., 2015). People engaging in socially deviant behaviors might reveal as criminals, but sometimes maintain undiscovered via exhibiting a high level of functioning in daily life.

A prominent overarching conceptualization of socially undesirable personality traits was proposed by Paulhus and Williams (2002) known as the Dark Triad of Personality (DT). It integrates (subclinical) narcissism, Machiavellianism and (subclinical) psychopathy as personality traits, hence, as dimensional constructs (Paulhus and Williams, 2002). All three DT traits have a personality structure in common where the motive to elevate the self while derogating others is predominant. Specifically, narcissism is characterized by feelings of grandiosity and striving for affirmation and reinforcement of the self. Machiavellianism involves the tendency toward manipulative and strategically calculated behaviors, with the focus on exploitation for the sake of personal gain, as well as cold affect (Paulhus and Williams, 2002). Psychopathy is described by deficits in affect and self-control, emotional indifference and impulsivity (Paulhus and Williams, 2002). The three traits and especially Machiavellianism and psychopathy show substantial intercorrelations, with the latter often exceeding *r* > 0.50 (Furnham et al., 2013). Research literature on gender differences in the DT traits has indicated that men robustly score higher on each of the DT traits. This gender effect has been shown in both, studies assessing each trait with separate questionnaires (e.g., Paulhus and Williams, 2002) and studies assessing the three traits simultaneously with the same inventory (e.g., Short Dark Triad Scale; Jones and Paulhus, 2014).

A wealth of previous studies examined the associations between the DT traits and other personality constructs, with some studies suggesting a “dark core” possibly reflecting shared underlying constructs (Book et al., 2015; O’Boyle et al., 2015; Bertl et al., 2017; Muris et al., 2017). In support of this assumption, several studies demonstrated convergent sanctions of the three DT traits with prominent, normative personality models including the Big Five (Goldberg, 1981) and the HEXACO model (Lee and Ashton, 2004). Within these personality models specifically Agreeableness and Honesty-Humility have been shown negatively associated with each DT trait (Lee and Ashton, 2005; Jakobwitz and Egan, 2006; Book et al., 2015; O’Boyle et al., 2015; Muris et al., 2017; Watts et al., 2017). In addition, interpersonal antagonism (Furnham et al., 2013) and lack of empathy or, more generally, callous affect (Furnham et al., 2013; Paulhus, 2014; O’Boyle et al., 2015) might represent shared underlying elements of the DT traits. Empirical support for this notion was given in many studies demonstrating substantial negative associations between the DT traits and various measures of socio-emotional abilities (e.g., Barlow et al., 2010; Howe et al., 2014; Jauk et al., 2016).

Similarly, many distinctive features with regards to the DT have been identified. As the DT traits show positive intercorrelations, it is essential to apply analyses controlling for shared variance of the three traits to identify independent contributions of each (Furnham et al., 2013). Impulsivity is considered a significant feature distinguishing psychopathy from Machiavellianism (Paulhus and Williams, 2002; Paulhus, 2014; Miller et al., 2017; Vize et al., 2018). In line with this, Machiavellianism has been associated with stronger resistance to temptations compared to psychopathy (Williams et al., 2010), while the latter has been clearly characterized by weak self-control (Jonason and Tost, 2010), stronger impulsive short-term mating strategies (Jonason et al., 2009; Jones and Paulhus, 2011a) and positive links with various measures of impulsivity. However, findings surrounding impulsivity and Machiavellianism are not always consistent (Williams et al., 2010; Jones and Paulhus, 2011b; Malesza and Ostaszewski, 2016; Miller et al., 2017; Vize et al., 2018). Apart from impulsivity, evidence indicates that psychopathy has the most aggressive nature. It has been most strongly related to various forms of aggression, such as physical aggression (Jones and Neria, 2015), cyber-aggression (Pabian et al., 2015) and bullying behaviors (Baughman et al., 2012). Machiavellianism, in contrast, constitutes a positive predictor of hostility (Jones and Neria, 2015). Examinations on reactive behaviors following physical or significant ego-threat furthermore revealed that psychopathy is associated with aggressive responses to physical threat, while ego-threat triggers reactive behaviors in narcissism (Jones and Paulhus, 2010). Apart from that, narcissism appears to be the only trait within the DT framework that has, to some degree, been regarded as adaptive under certain circumstances. Individuals with higher narcissism show more positive affiliative humor (Veselka et al., 2010; Martin et al., 2012), are perceived as popular at first glance by unacquainted peers (Back et al., 2010) and receive positive ratings in simulated job interviews (Paulhus et al., 2013), hence are making good first impressions. Furthermore, only narcissism, but neither of the other two DT factors, have been related to higher mental toughness (Papageorgiou et al., 2017, 2019) and lower stress perception (Papageorgiou et al., 2019).

With regards to emotional abilities, it has been argued that the DT traits share callousness as common underlying element. Emotional intelligence (EI) generally encompasses a set of skills related to the detection, processing and usage of affect-related information, such as one’s own or others’ emotions. It can be conceptualized and assessed via self-report (trait EI) or in tests on maximum performance (ability EI) (Salovey and Mayer, 1990; Petrides and Furnham, 2003; Mayer et al., 2016). The perception and recognition of other’s emotional expressions have been described as basal components of ability EI (Mayer et al., 2016). They are of high relevance in social situations, as they allow for adequate reactions and appropriate forms of social interaction (Bruce and Young, 1986; Haxby et al., 2000). In line with this, it has been shown that accurate emotion recognition and labeling are related to various positive outcomes. They constitute positive predictors of social skills, cooperative behaviors, and academic achievement, while they negatively predict conduct problems (Izard et al., 2001). The predominant view on emotion recognition ability as positive social resource suggests negative associations with socially maladaptive personality traits such as the DT. In line with this, a substantial amount of studies reported poor performance in emotion recognition measures, such as facial expression detection or social-perceptual Theory of Mind, across individuals with higher DT scores (Ali and Chamorro-Premuzic, 2010; Lyons et al., 2010; Dawel et al., 2012; Vonk et al., 2013, 2015; Konrath et al., 2014; Stanković et al., 2015). Together these previous findings suggest deficient emotion recognition ability in individuals with higher DT scores and confirm the common understanding of the three traits as “callousness constellation” (Paulhus, 2014). Of note is that the findings for narcissism appear not as consistent as for the other two DT traits. For instance, Wai and Tiliopoulos (2012) found a positive relation with the recognition of angry faces and Konrath et al. (2014) identified exploitativeness (as facet of narcissism) as positive predictor of the recognition of negative emotions. Also, a recent meta-analytic investigation revealed that only Machiavellianism and psychopathy negatively correlated with both ability and trait EI (Miao et al., 2019). This again indicates the occasional adaptive nature of narcissism and is in line with the conclusion, that narcissism represents the lightest trait among the DT (Rauthmann and Kolar, 2012).

The literature on negative associations between the socially aversive DT traits and emotional abilities supports the notion of the latter being desirable for interpersonal interactions. Therefore, one could assume that higher emotional ability may promote socially appropriate behaviors and inhibit antisocial behaviors. However, some evidence points to the opposite direction, where (high) emotional abilities may foster a broad span of social behaviors, among which not all might be appreciated (Austin et al., 2007; Côté et al., 2011). More specifically, some studies focused on the antisocial orientation of emotional abilities. Among these, it has been shown that women with higher social intelligence report more relational aggression (e.g., spread rumors, gossip, and social exclusion) (Loflin and Barry, 2016). Bacon and Regan (2016) also found a gender effect, where emotionally intelligent women reported higher general delinquency (e.g., vandalism, robbery) and interpersonal forms of delinquency (e.g., bullying, spreading rumors, and social exclusion) as compared to women scoring low on the measure of EI. Another study has shown positive relations between social cognition and bullying in children, whereas especially strong effects were shown for those taking the role of the leader bully (Sutton et al., 1999).

Previous findings therefore indicate that emotional abilities may also relate to harmful outcomes, which contradicts the perception of them as being generally desirable. It could be assumed that emotional abilities foster potential harmful interpersonal behavior only under certain circumstances or under some personality constellation. More specifically, they may be strategically used by people with antisocial dispositions who are trying to make use of their emotional skills for personal benefit or enjoyment. In line with this assumption it has been argued that the presence of (above-)average emotional abilities in antisocial personalities might promote even more harmful behaviors (Buss and Chiodo, 1991; Côté et al., 2011; Nagler et al., 2014). However, research addressing the potential consequences of high EI in individuals with socially deviant personality traits is rare. Côté et al. (2011) showed that Machiavellianism was associated with interpersonal deviant behavior (“I publicly embarrassed someone at work,” Bennett and Robinson, 2000) only when subjects had effective emotion regulation strategies (ability EI). Further supporting evidence was given in a study investigating the interaction effect of the DT traits and self-reported socio-emotional abilities (trait EI) on emotionally manipulative tendencies. It was shown that the positive interaction effect of both narcissism and psychopathy with emotional control (e.g., regulation of emotional expressions) predicted higher scores on manipulative tendencies. Further, psychopathy positively predicted manipulative tendencies along with high emotional sensitivity (toward other’s feelings) (Nagler et al., 2014). The phenomenon, where the interaction between dark personality traits and emotional abilities positively predicts socially deviant behaviors has also been referred to as dark EI (Nagler et al., 2014) and has been subject of only a few studies. Also, it has rarely been investigated by means of ability-based measures. The first aim of the present study is therefore to examine whether findings on dark EI can be applied to the ability to recognize emotions.

The second aim of the present study is to overcome two general problems surrounding psychological research, namely (i) the inability to replicate many psychological findings and (ii) the predominant use of Western samples. Psychological studies predominantly involve WEIRD samples; however, most people do not belong to the *W*estern, *E*ducated, *I*ndustrialized, *R*ich, and *D*emocratic societies (Henrich et al., 2010). It thus remains unclear whether the abovementioned findings on dark EI can be generalized onto other cultures. In this context, investigating effects in samples from countries strongly differing on cultural dimensions, as proposed by Hofstede (2011), is of particular interest, as similar result patterns across culturally diverse samples may point to globally valid effects (Montag, 2018). For this reason, the present study aims at independently replicating study findings in Germany (a Western country) and China (an Eastern country). Extant literature already points at cross-cultural, universal components of human nature found in both, Western countries and China (McCrae et al., 1996; McCrae and Costa, 1997; Montag and Becker, 2018). With regards to dark personality traits, Chinese translations of the Narcissistic Personality Inventory (Kwan et al., 2009; Cai et al., 2012), the Kiddie Machiavellianism Scale (Geng et al., 2016) and three different psychopathy scales (Levenson et al., 1995; Neumann et al., 2012; Shou et al., 2016, 2017) received good empirical support. Recently, a Chinese version of the Short Dark Triad assessing all three DT traits was also evaluated in a Chinese sample and showed psychometric properties comparable to the English original (Zhang et al., 2019). Apart from the mere transfer of inventories to China, some studies with similar designs have already been conducted to independently replicate findings in both Germany and China. Thereby, consistently comparable findings regarding interindividual differences in personality traits (Montag and Panksepp, 2017; Sindermann et al., 2018b) and genetic associations with personality traits (Montag et al., 2017; Sindermann et al., 2018a) were found in both German and Chinese samples.

The findings on cross-cultural replicability of personality taxonomies and study effects are guiding in terms of the generalizability and universality of certain personality traits and allow traits recognized in Western countries to be studied in Chinese samples (McCrae et al., 1996). Taken together, especially studies including samples of different cultures are needed. Given the deficient reproducibility of psychological findings as revealed by the Open Science Collaboration (2015), replications of study findings both within the same cultural area and beyond are of high interest, whereas the latter could enable additional assumptions about the transferability of study findings to other cultural groups. Cross-cultural psychological research is of high importance to recognize globally valid effects and cultural features in psychological personality profiles (Montag, 2018).

The present work follows two main aims. For the purpose of further investigating the interpersonal nature of the DT traits, we first aim at examining the construct of dark EI using a behavioral measure of emotional abilities. More specifically, we aim at studying the interaction effects of each DT trait with emotion recognition performance in predicting emotionally manipulative tactics. Secondly, we aim at contributing to cross-cultural research by conducting our study in two countries typically considered culturally different (Hofstede, 2011): Germany and China. We therefore strive to independently replicate our findings to derive potentially globally valid effects (Montag, 2018). At the same time, we aim at exploring cultural specificities concerning socially aberrant personality traits. Therefore, we will also investigate the correlations between all variables of interest. Eventually, the findings obtained from the present study should add knowledge to the debate on the empirical overlap of the DT traits.

## Materials and Methods

A survey including the variables of interest and other questionnaires (see Supplementary Data 1 and Supplementary Table 1 for further information) was accessible online between April and June 2018. Data collection took place at Ulm University, Germany and Beijing University of Civil Engineering and Architecture, China. Inclusion criteria were absence of psychiatric or neurological conditions and native speaker level in German or Mandarin Chinese, respectively.

### Participants and Procedure

The final German sample consisted of *N* = 198 participants (130 females, 68 males; age: *M* = 23.40, *SD* = 5.88, range = 18–57; 87.9% students) and the final Chinese sample of *N* = 223 subjects (105 females, 118 males; age: *M* = 19.01, *SD* = 1.06, range = 18–24; 100% students). As the participants would rate pairs of eyes of Asian and Caucasian models as part of the Eyes Test, they were additionally asked about the frequency to which they have experience with the other culture in daily life on a five-point Likert-type scale (*very rarely* to *very frequently*). Chinese participants (*M* = 2.52, *SD* = 1.17) had more everyday contact with the Western culture than German subjects had with the Chinese culture (*M* = 1.57, *SD* = 0.91; *U* = 11598.50, *p* < 0.001). 14.8% of Chinese participants (*N* = 33) reported to have visited a Western country, while 8.1% of German subjects stated to have visited China (*N* = 16). All study participants gave informed electronic consent (approved by the ethical review board of the University of Electronic Science and Technology of China, Chengdu, China) before completing the survey.

### Measures

#### Translations

At the time of data collection, only original English versions of the Short Dark Triad Scale and the Emotional Manipulation Scale were available. Therefore, these were translated to German and Mandarin Chinese. To make sure that the translated items reflected the meaning of the original English items, translations were performed in accordance with the guidelines for cross-cultural research (Brislin, 1970; Hambleton, 2001) and as recommended by the International Test Commission (Hambleton, 2001): First, questionnaires of interest were forward-translated from the source language (English) into the target language (German and Mandarin Chinese) by bilingual German and Chinese native speakers with previous experience in psychological test translations. In each sample, a second, independent bilingual person back-translated the resulting version into English. Differences between the original and the back-translated questionnaire were discussed until agreement regarding the equivalence of both questionnaires was reached. The German and Chinese translations can be obtained from the first author upon request. McDonald’s omega coefficients as internal reliability measure are depicted in Table 1 alongside descriptive statistics.

**TABLE 1:** Descriptive statistics of the scales of interest in Germany and China.

	*N*	*M*	*SD*	*Min*	*Max*	ω

German Sample						
Narcissism	198	2.60	0.53	1.11	4.00	0.68
Machiavellianism	198	2.94	0.61	1.44	4.78	0.76
Psychopathy	198	2.09	0.57	1.11	3.56	0.73
Emotional Manipulation	198	3.02	0.58	1	4.60	0.85
Eyes Test overall	198	0.65	0.10	0.24	0.85	0.81
Eyes Test Caucasian	198	0.69	0.12	0.19	0.92	0.69
Eyes Test Asian	198	0.60	0.11	0.14	0.86	0.67

**Chinese Sample**						

Narcissism	223	2.94	0.44	1.78	4.22	0.59
Machiavellianism	223	3.26	0.58	1.67	5.00	0.79
Psychopathy	223	2.34	0.55	1.11	3.89	0.69
Emotional Manipulation	223	3.05	0.59	1.27	5.00	0.89
Eyes Test overall	223	0.65	0.12	0.18	0.83	0.85
Eyes Test Caucasian	223	0.60	0.13	0.17	0.83	0.68
Eyes Test Asian	223	0.70	0.14	0.17	0.92	0.77

*Only 15 Items of the 16-item Emotional Manipulation Scale were included in the statistical analyses since the third item of the Chinese translation did not reflect the meaning of the English original item (please also see footnote 1). Eyes Test overall refers to the recognition of emotions from Asian and Caucasian pairs of eyes.*

#### Short Dark Triad Scale

Individual differences in the DT traits were assessed using the Short Dark Triad Scale (SD3; Jones and Paulhus, 2014). The 27-item scale operationalizes each trait according to its core facets: narcissism deals with perceived grandiosity and strive for ego-reinforcement (“I know that I am special because everyone keeps telling me so”) and Machiavellianism addresses strategic long-term orientation and manipulative attributes (“Avoid direct conflict with others because they may be useful in the future”), while psychopathy reflects impulsivity, antisocial behavior and callous affect (“People who mess with me always regret it”). Each DT component is measured with 9 items being answered on a five-point Likert-type scale (*disagree strongly* to *agree strongly*).

#### Emotional Manipulation Scale

The Emotional Manipulation (Austin et al., 2007) Scale was used to measure emotionally manipulative tactics. It assesses self-reported manipulation abilities one-dimensionally (e.g., “I can use my emotional skills to make others feel guilty”). Thus, the scale assesses the ability to behave in an emotionally manipulative manner, which we here label emotionally manipulative tactics. We applied the full 16-item scale. Comparisons of both the 10-item (as used in Nagler et al., 2014) and 16-item versions indicated higher internal reliability of the latter in both samples. A significant mistake in the Chinese translation of the third item was recognized after data collection had already started. We therefore decided to exclude this item in both samples for analyses to maintain comparability and facilitate replication^1^. The 15-item versions still showed considerable internal reliabilities (Table 1). Items were rated on a five-point Likert-type scale (*strongly disagree* to *strongly agree*).

#### Eyes Test

For the identification of emotions from the face, it has been shown that the dynamic or varying parameters of the face, such as the mouth and eye area, are of particular diagnostic value (e.g., Wegrzyn et al., 2017). Therefore, emotion recognition, which is considered a basal facet of social and emotional intelligence, was assessed with the revised adult version of the Eyes Test (Baron-Cohen et al., 2001) in German and Mandarin Chinese. The Eyes Test constitutes a forced choice paradigm, measuring the ability to recognize emotions or complex mental states of another individual from the eye area only (Oakley et al., 2016). Participants were presented with black-and-white images of pairs of eyes, displaying different types of mental states. For each image, subjects were instructed to choose one out of four words best describing the depicted state as quickly as possible. One of the adjectives constituted the correct answer, while three were incorrect. The number of correct assignments of an emotion word to the depicted state was measured. To enable a culturally fair measurement as suggested by, e.g., Triandis and Suh (2002), both Caucasian (Baron-Cohen et al., 2001) and Asian^2^ sets of pairs of eyes were presented in both samples. Participants were therefore asked to rate a total of 72 images (36 images for each version). The number of female and male stimuli was balanced. The order of images shown to all subjects was fixed, whereby the presentation of images depicting Caucasian, Asian, female and male pairs of eyes was balanced.

### Statistical Analyses

Data analysis was performed using IBM SPSS Statistics version 26 (IBM Corp, 2019). McDonald’s omega reliability coefficients were computed using JASP version 0.11.1 (JASP Team, 2019). After recoding of inverted items, (sub)scale means were computed for the SD3 and Emotional Manipulation Scale. Higher values on each (sub)scale indicate higher scores on the underlying construct. For the calculation of accuracy in the Eyes Test, items were first dichotomized (1 = correct answer, 0 = wrong answer) and means were calculated to create an overall performance score. Separate scores were calculated for Caucasian and Asian stimuli as well as positively, negatively and neutrally valenced states in accordance with Harkness et al. (2005). As a result, mean accuracy for the overall as well as the domain-specific (culture, valence) score could range between 0 and 1. The correlations between the German and Chinese (sub)samples were compared using Fisher’s z-tests. Analyses on measurement invariance will be addressed in the discussion part and were performed using R version 3.5 (R Core Team, 2017) and the R-package lavaan (Rosseel, 2012). Moderation models for the analysis of dark EI were calculated using the PROCESS macro for SPSS (Hayes, 2017).

## Results

### Descriptive Statistics

The descriptive statistics of the scales of interest are depicted in Table 1. The descriptive statistics for all variables under investigation can be obtained from Supplementary Table 1.

Most scales were not normally distributed (Supplementary Table 2). Age was significantly correlated with emotionally manipulative tactics in the German sample (*r* = −0.19, *p* = 0.006) and with overall Eyes Test performance (*r* = −0.16, *p* = 0.017), as well as emotion recognition of Asian (*r* = −0.14, *p* = 0.039) and Caucasian (*r* = −0.16, *p* = 0.018) pairs of eyes. Also, the DT traits showed partly substantial intercorrelations (Germany/China: narcissism – Machiavellianism ρ = 0.17/0.13 with *p* < 0.05; narcissism – psychopathy ρ = 0.25/0.26 with *p* < 0.001; Machiavellianism – psychopathy ρ = 0.48/0.40 with *p* < 0.001). Even though most of the aforementioned effect sizes can only be considered low to moderate (Cohen, 1988), statistical analyses were performed with the respective other DT traits and age controlled as covariates. Therefore, associations between scales were calculated by means of partial Spearman’s rank correlation coefficients. This statistical procedure was implemented for both the German and Chinese (sub)samples to maintain consistency across samples. To control for multiple testing artifacts, manual Bonferroni corrections for six correlations per cultural/gender group (α ≤ *p*/6 = 0.05/6 = 0.008) were performed.

### Cultural and Gender Differences

The scores on all DT traits were higher in the Chinese as compared to the German sample (all *p* < 0.001). Within each culture, males scored significantly higher than females did on Machiavellianism (*M* = 3.30, *SD* = 0.58 vs. *M* = 2.76, *SD* = 0.55, *U* = 2155.50, *p* < 0.001 in Germany; *M* = 3.37, *SD* = 0.60, *M* = 3.14, *SD* = 0.55, *U* = 4835.00, *p* = 0.005 in China) and psychopathy (*M* = 2.32, *SD* = 0.47 vs. *M* = 1.98, *SD* = 0.58, *U* = 2685.50, *p* < 0.001 in Germany; *M* = 2.46, *SD* = 0.57 vs. *M* = 2.20, *SD* = 0.49, *U* = 4646.00, *p* = 0.001 in China). The scores on narcissism neither differed in Germany (*p* = 0.47) nor China (*p* = 0.18) between the genders. With regards to emotionally manipulative tactics, mean scores were equal between Germany and China (*p* = 0.63), but higher in males compared to females, respectively (*M* = 3.18, *SD* = 0.51 vs. *M* = 2.95, *SD* = 0.60, *U* = 3209.00, *p* = 0.002 in Germany; *M* = 3.15, *SD* = 0.62 vs. *M* = 2.94, *SD* = 0.54, *U* = 4721.00, *p* = 0.002 in China).

Overall Eyes Test performance was equally pronounced in Germany and China (*p* = 0.064). However, clear ingroup advantages became apparent – that is, German subjects were superior in recognizing emotions from Caucasian pairs of eyes (*p* < 0.001), while the reverse applied to the Chinese sample (*p* < 0.001). Among German subjects, females (*M* = 0.71, *SD* = 0.11) were more accurate than males (*M* = 0.65, *SD* = 0.13, *U* = 3246.50, *p* = 0.002) in the Eyes Test including Caucasian pairs of eyes, while recognition of Asian pairs of eyes did not differ between German females and males (*p* = 0.147). In the Chinese sample, females performed better in recognizing emotions from Asian (*M* = 0.73, *SD* = 0.12 vs. *M* = 0.68, *SD* = 0.15, *U* = 4800.00, *p* = 0.004) and Caucasian (*M* = 0.62, *SD* = 0.11 vs. *M* = 0.58, *SD* = 0.14, *U* = 5230.00, *p* = 0.044) pairs of eyes as compared to males. Eyes Test performance for the respective other culture did not differ between the German and Chinese sample (*p* = 0.669). However, Eyes Test performance for the own culture was better in the Chinese sample (*U* = 19352.00, *p* = 0.028). Additionally, Eyes Test performance for Caucasian eyes was positively associated with the degree of experience with Western culture in the Chinese group (ρ = 0.29, *p* < 0.001). The reverse was not true for the German sample (ρ = −0.04, *p* = 0.578) (also see Supplementary Tables 3–6).

Due to gender differences, effect sizes were calculated separately for females and males in Germany and China. In addition, given (i) the higher degree of everyday contact with the Western culture and (ii) its significantly positive relationship with the Eyes Test performance for Caucasian pairs of eyes as well as (iii) the superior performance in the Eyes Test of the own culture in the Chinese sample, and in order to (iv) increase concurrency and maintain culturally fair measurements, Eyes Test performances were subsequently analyzed only for the corresponding culture.

### Correlations

Results for the correlations of interest are depicted in Table 2.

**TABLE 2:** Partial rank-correlation coefficients for the variables of interest and the DT traits (Spearman’s ρ), controlled for the respective other two members of the DT and age.

German Sample	Females *N* = 130			Males *N* = 68		
		
	Narcissism	Machiavellianism	Psychopathy	Narcissism	Machiavellianism	Psychopathy
Emotional Manipulation	0.20*	0.37**	0.12	0.21	0.34**	0.19
Eyes Test Caucasian	–0.10	0.09	–0.16	0.07	–0.16	–0.22

**Chinese Sample**	**Females *N* = 105**			**Males *N* = 118**		
		
	**Narcissism**	**Machiavellianism**	**Psychopathy**	**Narcissism**	**Machiavellianism**	**Psychopathy**

Emotional Manipulation	0.14	0.38**	0.25*	0.05	0.36**	0.22*
Eyes Test Asian	0.16	–0.13	–0.17	0.09	0.14	–0.15

*p < 0.05; **p < 0.01, two-tailed. Numbers in bold indicate the effect sizes surviving Bonferroni-correction (α ≤ *p*/5 = 0.05/6 = 0.008). The correlations for all the assessed variables including the correlations between the DT traits and the Eyes Test performance for the respective other culture can be obtained from Supplementary Table 7. Corresponding information on Fisher’s z-tests can be obtained from Supplementary Data 2. Only 15 Items of the 16-item Emotional Manipulation Scale were included in the statistical analyses since the third item of the Chinese translation did not reflect the meaning of the English original item (please also see footnote 1).

#### Emotionally Manipulative Tactics

Machiavellianism showed the most consistent and strongest correlations with emotionally manipulative tactics, with equal effect sizes in all subsamples (all *z* < |0.29|, *p* > 0.05). Positive correlations were also found for psychopathy in the Chinese sample for both males and females and were of same strength (*z* = |0.23|, *p* = 0.82) and for narcissism in German females. However, these effects did not survive Bonferroni correction while effects for Machiavellianism remained robust.

#### Emotion Recognition

Impairment in emotion recognition ability was moderately apparent in psychopathy, however, correlations showed no statistical significance. Correlations were equally pronounced in Chinese and German females (*z* = |0.08|, *p* = 0.94) and males (*z* = |0.47|, *p* = 0.64). The correlation patterns with the other two DT traits were inconsistent and also non-significant across samples.

### Moderation Models/Dark EI

In order to investigate the relationship between the DT traits and emotionally manipulative tactics at different levels of emotion recognition ability, the DT traits were considered independent variables within the PROCESS macro for SPSS (Hayes, 2017), while emotion recognition performance was put as moderator variable and emotionally manipulative tactics as dependent variable. Moderation models were calculated for overall Eyes Test performance – i.e., Eyes Test performance across all valences – as moderator variable first. In case of (marginally) significant interaction effects, subsequent valence-specific analyses were performed. The results refer to the score of emotionally manipulative tactics when emotion recognition was high (*M*+1*SD*), average (*M*) and low (*M*−1*SD*). Additional simple slope analyses (pairwise contrasts) were calculated to investigate the conditional effects at different scores on the Eyes Test. Moderation models were calculated separately for gender and culture. Moreover, the remaining two DT traits and age were included as covariates (see also section “Descriptive Statistics”). Data for the German sample were again analyzed with regards to the performance on the Eyes Test for Caucasian pairs of eyes and vice versa for the Chinese sample (see also section “Cultural and Gender Differences”). Subsequently, significant moderation models (*p* ≤ 0.05) will be presented.

#### Narcissism

**FIGURE 1:**
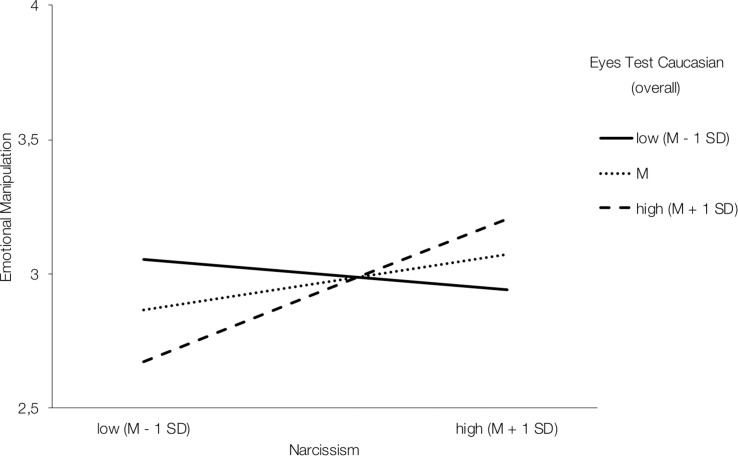
Interaction effect of narcissism and overall emotion recognition performance in predicting emotionally manipulative tactics in the German female sample.

In the German female sample, overall emotion recognition ability positively moderated the association between narcissism and emotionally manipulative tactics [*B* = 2.73, *SE* = 0.83, *F*(1,123) = 10.76, Δ*R*^2^ = 0.05, *p* = 0.001, 95% CI (1.08; 4.37)] (Figure 1). Simple slope analyses indicated a non-significant effect for weak (*M*−1*SD*) emotion recognition ability (*p* = 0.424), but a significant effect for medium (*M*) [*B* = 0.19, *SE* = 0.08, *p* = 0.02, 95% CI (0.03; 0.36)] and high (*M*+1*SD*) [*B* = 0.50, *SE* = 0.11, *p* < 0.001, 95% CI (0.27; 0.72)] emotion recognition ability.

Further investigating valence-specific effects, we observed significant positive interactions of narcissism and the recognition of neutral (*p* = 0.008) and negative (*p* = 0.029) emotional expressions in predicting emotionally manipulative tactics. Again, pairwise contrasts showed strongest pronounced effects (each *p* < 0.001) when emotion recognition ability was above average (Supplementary Figures 1A,B). This pattern of effects could not be observed in the German male sample or the Chinese female or male sample (all *p* > 0.05).

#### Psychopathy

**FIGURE 2:**
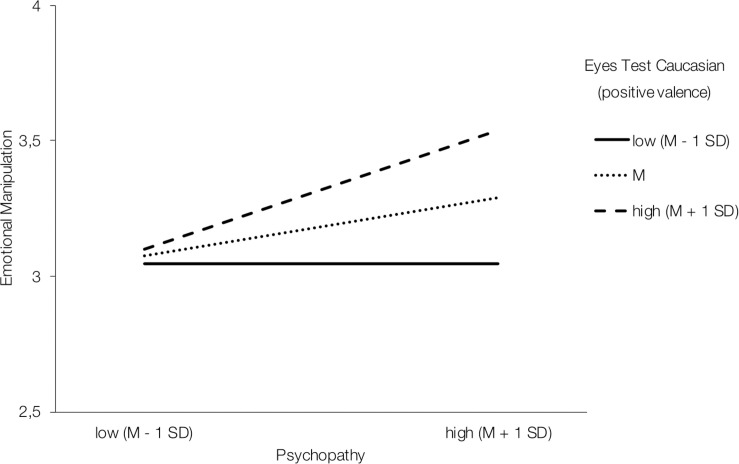
Interaction effect of psychopathy and positive emotion recognition performance in predicting emotionally manipulative tactics in the German male sample.

In the German male sample, overall emotion recognition ability showed a marginally significant interaction effect with psychopathy in predicting emotionally manipulative tactics [*B* = 1.57, *SE* = 0.81, *F*(1,61) = 3.80, Δ*R*^2^ = 0.04, *p* = 0.056, 95% CI (−0.04; 3.18)]. Simple slope analyses showed non-significant effects when Eyes Test performance was low (*p* = 0.794), but significant effects for medium [*B* = 0.25, *SE* = 0.12, *p* = 0.049, 95% CI (0.002; 0.49)] and especially high emotion recognition ability [*B* = 0.45, *SE* = 0.16, *p* = 0.008, 95% CI (0.12; 0.78)] (Supplementary Figure 1C). In terms of valence-specific effects, positive emotion recognition ability [*B* = 1.35, *SE* = 0.57, *F*(1,61) = 5.57, Δ*R*^2^ = 0.06, *p* = 0.021, 95% CI (0.21; 2.49)] positively moderated the association between psychopathy and emotionally manipulative tactics. Interestingly, simple slope analyses showed that the effect was driven by high emotion recognition ability only [*B* = 0.46, *SE* = 0.17, *p* = 0.007, 95% CI (0.13; 0.79)], whereas the effects of weak (*p* = 0.997) and medium (*p* = 0.068) Eyes Test performance were not significant (Figure 2).

The here shown interaction effects of psychopathy and (positive) emotion recognition ability were neither pronounced in the German female sample, nor in the Chinese male or female sample (all *p* > 0.05).

#### Machiavellianism

**FIGURE 3:**
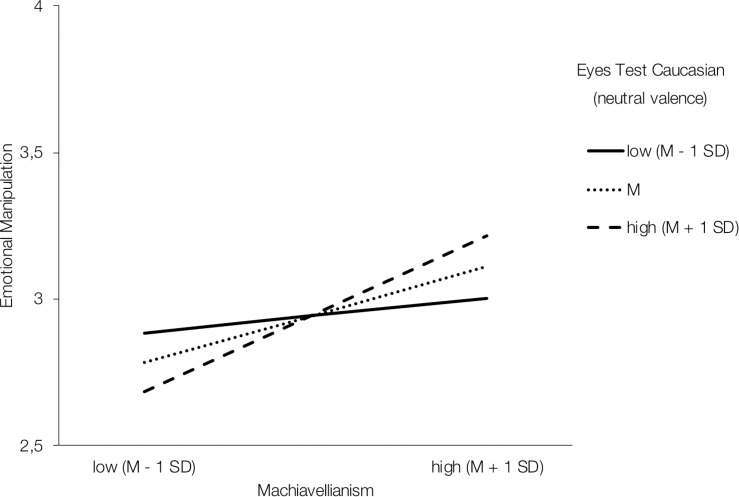
Interaction effect of Machiavellianism and neutral expression recognition performance in predicting emotionally manipulative tactics in the German female sample.

Regarding Machiavellianism, marginally significant interaction effects with overall Eyes Test performance were found in the German female sample in the prediction of emotionally manipulative tactics [*B* = 1.54, *SE* = 0.78, *F*(1,123) = 3.93, Δ*R*^2^ = 0.02, *p* = 0.05, 95% CI (0.003; 3.07)]. Investigating simple slopes, the conditional effect for low emotion recognition ability showed no statistical significance (*p* = 0.248). However, the effects were strongly pronounced at medium [*B* = 0.33, *SE* = 0.09, *p* < 0.001, 95% CI (0.15; 0.50)] and high [*B* = 0.50, *SE* = 0.11, *p* < 0.001, 95% CI (0.28; 0.72)] levels of Eyes Test performance (Supplementary Figure 1D). Valence-specific models showed that the interaction effect was also present for the recognition of neutral expressions [*B* = 1.30, *SE* = 0.60, *F*(1,123) = 4.74, Δ*R*^2^ = 0.02, *p* = 0.031, 95% CI (0.12; 2.48)]. Concerning conditional effects, weak ability to recognize neutral expressions showed no significant effect (*p* = 0.449) while both, average [*B* = 0.30, *SE* = 0.09, *p* = 0.002, 95% CI (0.11; 0.48)] and above average performance [*B* = 0.48, *SE* = 0.11, *p* < 0.001, 95% CI (0.27; 0.70)] in recognizing neutral expressions showed significant effects on the relationship between Machiavellianism and emotionally manipulative tactics (Figure 3).

In the Chinese female and male sample as well as the German male sample, there were no significant interaction effects of Machiavellianism and the overall Eyes Test performance.

## Discussion

The present study addressed two main topics concerning the DT traits. Given the ongoing discourse on the distinguishability of the DT traits, we aimed at further disentangling their interpersonal nature by examining dark EI by means of the Eyes Test assessing emotion recognition ability. Since personality psychology aims for cross-cultural replication studies and reproducibility of psychological findings, we conducted our study in both, a more Western and a more Eastern country, that are considered culturally different (Hofstede, 2011): Germany and China. In this manner, we were able to identify potentially globally valid effects and derive specific culture features.

As gender differences on the variables of interest have been previously demonstrated (e.g., Jones and Paulhus, 2014) and partially observed in our study, we calculated our data separately for males and females. Also, given the substantial DT intercorrelations found elsewhere (e.g., Muris et al., 2017) and, in parts, in our study, we included the respective other two DT traits as covariates in the statistical analyses. Since also age was a significant correlate of some scales of interest, it was considered as a covariate. Lastly, we included a culturally fair measurement with the Eyes Test as it included both Caucasian and Asian pairs of eyes.

### Summary and Interpretation of Findings

#### Correlates of the DT

As pointed out in the introduction part, some researchers emphasize the reduction of the DT traits onto a single underlying “dark core” (e.g., Bertl et al., 2017). Others, however, support the independent position of each trait (e.g., Jones and Paulhus, 2011a, 2014). In the present study, we were able to show robust, cross-culturally replicable positive associations with emotionally manipulative tactics for Machiavellianism only. In addition, a negative non-significant trend of associations with emotion recognition ability could only be observed for psychopathy across all (sub)samples. This result pattern was uniquely consistent for psychopathy, however, sets a trend only and should be interpreted cautiously. No cross-culturally remarkable relations with the variables of interest could be observed for narcissism. Given these differentially pronounced effects when controlling for shared variance among the DT constructs – which is of great importance when investigating their relationships with other variables (Furnham et al., 2013) – we add further evidence for the independent role of each DT trait. It can therefore be concluded that the assumption of each trait of the DT framework as unique is legitimate and superior over mapping it as single construct. This is further supported by our finding that the genders scored differentially on these traits. More specifically, it was cross-culturally shown that males scored significantly higher on Machiavellianism and psychopathy than females, while no genderwise differences were observed for narcissism. If all traits would measure the same underlying construct, however, it might be expected that males have higher scores across all DT traits.

#### Dark EI

Dark EI can be described as interplay between dark personality traits and emotional abilities leading to interpersonally deviant behaviors. In past research, positive interaction effects between DT traits and self-report trait EI were found to positively predict emotionally manipulative tendencies (Nagler et al., 2014), suggesting emotional skills to be a potential risk factor in DT traits. In the present study, we exploratively investigated whether emotion recognition performance moderates the relationship between the DT traits and emotionally manipulative tactics. Emotion recognition performance was measured by means of the Eyes Test, a forced choice paradigm, while DT traits and emotionally manipulative tactics were measured with self-report questionnaires. We found emotion recognition as significant moderator for the relationship between all DT traits and emotionally manipulative tactics. Among these, simple slope analyses consistently revealed that emotionally manipulative tactics were highest when subjects had both high emotion recognition and high DT scores. Even though we found significant interaction effects for all DT traits, results were highly gender- and culture-dependent. More precisely, in the sample of German females, this pattern of results could be observed for narcissism and overall emotion recognition and specifically for the recognition of neutral and negative emotions. In the sample of German males, this pattern was found for psychopathy and overall emotion recognition, though especially pronounced for the recognition of positive emotions. Furthermore, high scores on neutral expression recognition ability significantly moderated the relationship between Machiavellianism and emotionally manipulative tactics in the German female sample. None of the effects was robustly shown across cultures or genders.

Our results have various indications. Firstly, we could replicate earlier findings on dark EI for narcissism and psychopathy (Nagler et al., 2014) with a performance-based approach to measure emotional abilities. In addition, we also found Machiavellianism to interact with emotional abilities in predicting emotionally manipulative tactics. Therefore, we were able to show that all DT traits are gender- and culture-specifically associated with stronger emotionally manipulative tactics when emotional abilities are present and above average.

Generally, the DT traits are reported to be associated with low emotional skills as e.g., lack of empathy (Jonason et al., 2013; Konrath et al., 2014; Schimmenti et al., 2019), low ability and trait EI (e.g., Barlow et al., 2010; Jauk et al., 2016), weak emotion recognition ability (Lyons et al., 2010; Dawel et al., 2012; Wai and Tiliopoulos, 2012; Vonk et al., 2013, 2015; Stanković et al., 2015) and inappropriate emotional reactions (Ali et al., 2009). As mentioned above, emotional skills are often linked to positive social outcomes. As a consequence, it could be assumed that extant emotional abilities may protect individuals scoring high on DT traits from deviant interpersonal behaviors. However, as in the study of Nagler et al. (2014), our results indicate the contrary. Since emotionally manipulative tactics were consistently highest when both emotional abilities and scores on DT traits were (above-)average, we interpret high emotional abilities as a risk factor with the potential to promote rather than to prevent deviant behaviors in individuals with pronounced dark personality traits. Among the emotional skills, each DT trait appears to be associated with a valence-specific ability. Dark EI in narcissism could be observed for the overall recognition of mental states and was especially pronounced for both neutral and negative emotional expressions (German females), suggesting that narcissism associates with generally higher emotion recognition abilities. Dark EI in psychopathy, in contrast, was especially characterized by accurate recognition of positively valenced emotions (German males). Machiavellianism, however, was found to be linked to the recognition of mental states with neutral valence (German females), suggesting that these traits are linked to emotion-specific recognition abilities. Eventually, the findings concerning dark EI suggest that individuals scoring higher in either DT trait may tend to use emotional manipulation whenever it fits with the emotion recognition abilities they are particularly good at. In this context, (German) males with higher psychopathy might emotionally manipulate especially people in positive (e.g., playful, flirtatious) states. Similarly, (German) females with higher Machiavellianism may tend to emotionally manipulate people when they are displaying neutral, non-affective (e.g., interested, reflective) states. In contrast, (German) females scoring higher in narcissism may tend to emotionally manipulate other people regardless of their emotional states.

### Cultural Generalizability

By conducting the study in Germany and China, we aimed at identifying potentially globally valid effects while simultaneously working out culturally characteristic effects. In terms of dark EI, our results were highly specific to the German sample. Previous studies in the field of dark EI were conducted with Western samples only (Austin et al., 2007; Côté et al., 2011; Nagler et al., 2014; Bacon and Regan, 2016; Loflin and Barry, 2016). We are not aware of similar studies with subjects from Asian countries to date. As we could not find effects in the Chinese sample, it appears that emotional abilities may constitute additional risk factors for deviant behaviors in people scoring high on DT traits in Western cultures only. This finding might be traced back to cultural features generally observed in more Western and more Eastern cultures, that are described as individualism vs. collectivism. On the group-level, individualism prevails in Western and industrial countries, while collectivism predominantly characterizes Eastern cultures (Hofstede, 2011). Accordingly, individualism is rather prevalent in Germany, while collectivism is predominant in China. This cultural dimension refers to the degree to which individuals integrate themselves into groups: collectivistic cultures are generally characterized by community orientation and a strong connection with the ingroup. In individualist cultures, the independence and autonomy of the individual are of high importance and goals of the individual prevail, while interpersonal relationships are secondary (Hofstede, 2011). Therefore, one would expect emotional abilities to promote interpersonally kind and empathetic behaviors in a collectivistic culture instead of manipulative behaviors toward another individual. From our results, we can cautiously draw the conclusion that people with higher scores on either of the DT traits might systematically make use of their emotional skills in order to take advantage of (e.g., exploit) another individual. However, such behaviors might be more probable in a more individualistically characterized culture like Germany (see also discussion on agentic vs. communal narcissism in the discussion section “Measures”). At least, our data suggest that interpersonal deviant behavior in DT traits is rather independent of emotional abilities in the Chinese sample and they neither represent a risky nor protective factor for the use of emotionally manipulative tactics.

Also, further findings of the present study indicate culturally generalizable positive associations between Machiavellianism and emotionally manipulative tactics throughout all subsamples. Also, though not statistically significant, psychopathy was robustly characterized by low emotion recognition performances.

### Limitations and Outlook

Several limitations of the present study with regards to the sample, study materials and their psychometric properties should be mentioned.

#### Sample

Sample sizes of *N* = 198 in Germany and *N* = 223 in China were generally satisfying. However, as we decided to look at results separately for males and females, the subgroup sample sizes were much smaller (smallest sample with *N* = 68 for German males to largest sample with *N* = 130 for Chinese males). Additionally, the two samples from Germany and China were not perfectly matched with regards to, e.g., age and gender ratio. Also, the emotion recognition performance from Caucasian pairs of eyes in the German sample was inferior to the emotion recognition performance from Asian pairs of eyes in the Chinese sample. Taken together, both samples were not perfectly comparable and replication requirements were not fully met. However, finding similar correlation patterns across such diverse samples even strengthens the global validity of some of the findings. Nevertheless, it makes the interpretation of differences (as observed for dark EI) more difficult. Moreover, participants were mostly students, which limits the generalizability of the results.

#### Measures

The here applied Eyes Test is usually used in studies on Theory of Mind. However, the perception of emotions constitutes a basal facet of ability EI (Salovey and Mayer, 1990; Mayer et al., 2016). In addition, work by Oakley et al. (2016) indicated that the Eyes Test is more useful for emotion recognition than Theory of Mind research. Although the Eyes Test allows for the distinction of correct from incorrect answers (Baron-Cohen et al., 2001), it has been argued that the response alternatives given in the Eyes Test do not allow for a single unambiguous and true correct answer. However, in performance measures, there should only be a single correct besides one or more incorrect answer(s) (Wilhelm et al., 2014). In this context, it has also been shown, that many of the adjectives used in the Eyes Test are underrepresented in ordinary language (e.g., “contemplative”) and performance on the Eyes Test therefore highly depends on linguistic abilities (Olderbak et al., 2015). Even though typical for forced choice paradigms, the presentation of response options evokes emotional concepts. These concepts facilitate better performances compared to tasks, where emotions have to be assigned freely without a preselection of adjectives potentially describing the depicted state (Barrett, 2017). Furthermore, the ability to read emotions from the eye area only assesses a very specific facet of EI. In the Eyes Test, subjects are instructed to answer as quickly as possible. However, in our study design, we could not ensure that subjects would respond within a certain time frame. Given that affective intuitions predominantly include automatic processes (Greene and Haidt, 2002), including a time restriction or measuring reaction times could have promoted quick and automatic responses. The here described disadvantages indicate, that performance on the Eyes Test cannot be equated ability EI. A replication of the present findings with reliable and valid tools for assessing the ability to recognize emotions from facial expressions (e.g., Wilhelm, 2005) and the eye area (Olderbak et al., 2015) are necessary. More importantly, a replication of the present findings with an established and broad ability EI measure such as the Mayer-Salovey-Caruso Emotional Intelligence Test (Mayer et al., 2002), covering different branches of EI, should be conducted.

Regarding the study of emotionally manipulative tactics, we used a self-report measure where subjects rate attitudes toward emotional manipulation and indicate whether they are able to behave in corresponding manners. In future studies it would be interesting to measure whether subjects actually showed manipulative behaviors in the past or whether they make use of their ability to behave manipulatively. The mere ability to show a certain behavior, or attitudes toward a certain behavior, do not necessarily equal actual behaviors. Aside from this, psychological manipulation covers a wide range of persuasive influences in different domains. Manipulative behaviors can have manifold causes and can be expressed in various ways (Hamilton et al., 1986; Buss et al., 1987; Kligman and Culver, 1992; Potter, 2006). The present findings, however, are limited to emotional manipulation (e.g., controlling other people’s behaviors by inducing guilt, shame or anxiety; Austin et al., 2007). Therefore, differential associations between the DT and other domains of manipulative behaviors, such as coercion, deceit, seduction or bribery, should be addressed in future studies. Not least because of the emotional nature of manipulative tactics investigated in the present work, a meaningful association with emotional abilities such as emotion recognition is more probable. It remains unclear whether and to what degree the here observed effects can also be applied to other forms of manipulation.

In the present study, we focused on a popular selection of dark personality traits (the DT). However, there are several more socially maladaptive traits that could extend this taxonomy (Boyle et al., 2015; Paulhus and Jones, 2015). Chabrol et al. (2009) observed that subclinical sadism positively relates to all DT traits, yet is sufficiently distinct to be incorporated into the so-called “Dark Tetrad” framework (Chabrol et al., 2009). Later work confirmed the validity of the Dark Tetrad of personality by showing that the subclinical sadistic trait is predictive of laboratory cruel behaviors beyond the other DT traits. The trait, which has been termed “everyday sadism” ever since, describes individuals experiencing enjoyment from harm caused to others in daily life. The trait covers pleasure from the direct infliction of harm (e.g., physically, verbally) and/or vicarious (e.g., brutal video games, movies) forms of harm (Buckels et al., 2013). Beyond that, Paulhus and Jones (2015) identified the potential of a construct referred to as Amoralism to add further diversity to the dark trait spectrum. Amoralism comprises a general disregard for other people and is driven by internal dispositions in three domains: hedonism and low impulse-control (Lascivia), low levels of frustration tolerance and personal dissatisfaction (Frustralia) as well as pleasure in brutality and other peoples’ suffering (Crudelia) (Paulhus and Jones, 2015; Mladenovic and Knezevic, 2018). Along with the traits just mentioned, six further personality traits (egoism, greed, moral disengagement, psychological entitlement, self-centeredness, and spitefulness) have been identified that are antagonistic in nature (Moshagen et al., 2020). Future (replication) studies should therefore include more traits of the broad dark personality spectrum in order to gain further insight into traits potentially involved in dark EI.

Beyond adding further traits, it would also be of great importance to investigate whether specific result patterns occur when investigating each DT trait on its facet-level. This would provide more insights into the aspects particularly involved in dark EI. The SD3, which was used here, represents a brief measure of the DT, where each trait is assessed only as broad factor without sub-facets. Therefore, application of the standard measures of the DT giving also insights into the facets of the traits, namely the Self-Report Psychopathy Scale (Williams et al., 2007), the Mach IV (Christie and Geis, 1970) and the Narcissistic Personality Inventory (NPI; Raskin and Hall, 1979), allowing for a more fine-grained assessment, would be of interest for replication studies [for an overview of available scales measuring the DT traits as single constructs or combined see Paulhus and Jones (2015)]. Concerning the conceptualization of narcissism, a distinction between agentic and communal components emerged. While the self-enhancing motives are present in both variants, individuals seek superiority in either agentic (e.g., “I am the most intelligent person I know”) or communal (e.g., “I am the most helpful person I know”) domains. Therefore, they satisfy their needs by different means (Gebauer et al., 2012). This distinction is of particular interest for cross-cultural designs including countries differing on the individualism – collectivism dimension. More specifically, seeking self-affirmation in agentic domains may oppose fundamental values of a rather collectivist culture like China (Hofstede, 2011; Cai et al., 2012; Gebauer et al., 2012), while it seems to better fit with more individualist cultures like Germany. With respect to the present study, this point may serve as an explanation for the non-replicability of findings on dark EI and narcissism. Therefore, a replication study including measures on agentic as well as communal narcissism is necessary.

#### Psychometric Properties

In order to conduct valid group comparisons, the requirement of measurement invariance should be fulfilled (Van de Schoot et al., 2012). It generally assesses whether a latent construct is psychometrically equivalent across groups. As we translated the SD3 and the Emotional Manipulation Scale on our own, we tested both scales with regards to measurement invariance in the German and Chinese sample. First, we investigated the factor structure by means of exploratory factor analysis (EFA). Additionally, we tested measurement invariance in a structural equation modeling framework using confirmatory factor analysis (CFA). For the SD3, we tested a correlated three-factor model, while for the Emotional Manipulation Scale, we tested a single-factor model. Invariance is tested stepwise, starting from configural invariance, which constitutes the least restrictive form and a prerequisite for stricter invariance testing steps. It indicates equivalent basic organization of latent constructs (in our data, e.g., 9 loadings on each of the DT factors). In other words, if configural invariance is fulfilled, an equivalent factorial structure of a measure is supported in two groups (Schwab and Helm, 2015; Putnick and Bornstein, 2016). Our analyses revealed that both the SD3 [χ^2^(*df*) = 1525.24 (642), *p* < 0.001, CFI = 0.65, RMSEA = 0.08, SRMR = 0.10] as well as the Emotional Manipulation Scale [χ^2^(*df*) = 655.03(180), *p* < 0.001, CFI = 0.78, RMSEA = 0.11, SRMR = 0.08] were configural non-invariant across the German and Chinese sample. Accordingly, the loading patterns of manifest items on latent constructs differed in Germany and China. Redefining the constructs by eliminating weak- or cross-loading items, thus using shorter forms, did neither improve model fit nor result in measurement invariance. A problem especially occurring in cross-cultural research is that the same term might lead to different conceptual understandings of the underlying construct, because the term is differently interpreted or connotated across cultures (Chen, 2008). Furthermore, characteristics of the sample (size) and the model may moderate invariance (Putnick and Bornstein, 2016). As addressed in the introduction part, many researchers questioned the distinctiveness of Machiavellianism and psychopathy. We also observed many cross-loadings of psychopathy items on the Machiavellianism factor and vice versa, supporting that the two constructs are closely intertwined. Summarizing, the SD3 and the Emotional Manipulation Scale were not equivalent across the two cultures. We recommend replication of the here presented results (i) using bigger samples and (ii) using scales showing good model fit in the respective languages. A validated German version of the SD3 has been recently published (Malesza et al., 2019) and a Chinese translation recently reached acceptable model fit (Zhang et al., 2019). However, we again would like to point out, that despite the group differences, we were able to observe comparable and often similar correlation patterns between the DT and the other scales of interest (see also Supplementary Table 7). Therefore, we were still able to demonstrate functional equivalence of the applied scales (Schmitt et al., 2007).

## Conclusion

The present study added knowledge to the current state of research surrounding the DT traits and their interpersonal correlates. For the first time the construct of dark EI has received empirical support using a performance-based approach to assess emotional abilities. However, the latter effect was exclusively found in the German sample, indicating culturally specific features. Furthermore, the present findings stress the relevance to consistently control for shared variance between the DT traits in order to investigate the unique contribution of each. Although measurement invariance could not be reached for two of the scales of interest across the nations, similar correlation patterns with other variables indicated functional equivalence. However, given some methodical limitations, further studies should consider alternative measures.

## Data Availability Statement

The datasets generated for this study are available on request to the corresponding author.

## Ethics Statement

The studies involving human participants were reviewed and approved by the Ethics Committee of the University of Electronic Science and Technology of China, Chengdu, China. The participants provided their electronic informed consent to participate in this study.

## Author Contributions

HS and CS designed the present study and collected the German data. HS drafted the present manuscript and conducted the statistical analyses. BB, CM, CS, KK, and YM gave helpful advices on how to improve the manuscript. YM provided the Eyes Test with Asian pairs of eyes. ML conducted the data collection in China. All statistical analyses were independently checked by CS. All authors revised and approved the final version of the manuscript.

## Conflict of Interest

The authors declare that the research was conducted in the absence of any commercial or financial relationships that could be construed as a potential conflict of interest.

## Footnotes

1 The third item did not reflect the meaning of the English original item. In the course of data collection, it was changed according to the source meaning. We compared scores on this item before and after modification and found out significant discrepancies. Therefore, the item was excluded in China. We also excluded it in Germany in order to meet criteria for independent replication.

2 http://brain.bnu.edu.cn/home/yinama/research.html
