# Supplementary material for: The Dark Side of Emotion Recognition – Evidence From Cross-Cultural Research in Germany and China
Source: Front Psychol. 2020 Jul 9;11:1132. doi: 10.3389/fpsyg.2020.01132 (PMC7363803; doi:10.3389/fpsyg.2020.01132)
Supplement: Supplementary file 1 [file Data_Sheet_1.pdf]

Supplementary Material

The Dark Side of Emotion Recognition – Evidence from Cross-Cultural Research in Germany and China

Helena S. Schmitt, Cornelia Sindermann, Mei Li, Yina Ma, Keith M. Kendrick, Benjamin Becker, Christian Montag

Supplementary Data 1. Description of Vengefulness scales

Next to the measures mentioned in the main manuscript, two measures were implemented to assess individual differences in vengefulness, which both were already available in validated German and Chinese translations (Sindermann, Luo, Zhao, Li, Li, Kendrick, Panksepp, & Montag, 2018b). The Transgression-Related Interpersonal Motivations Inventory (TRIM-12; (McCullough et al., 1998; McCullough & Witvliet, 2002) was used to measure event-related vengefulness, as it assesses attitudes toward a transgressor, that is, a person who caused hurt to an individual. Such attitudes are described on two facets: Revenge Motivation ("I want to see him/her hurt and miserable") is measured with five items and Avoidance Motivation ("I withdraw from him/her") with seven items. All items are answered on a five-point Likert-type format (*strongly disagree* to *strongly agree*), respectively. The Vengeance Scale (Stuckless & Goranson, 1992) represents a one-dimensional measure for general trait vengefulness ("Anyone who provokes me deserves the punishment that I give them"). The scale consists of 20 items, responded on a seven-point Likert-type scale (*strongly disagree* to *strongly agree*).

Supplementary Table 1. Descriptive statistics of Vengefulness scales in Germany and China

	N	*M*	*SD*	*Min*	*Max*	ω
**GERMAN SAMPLE**						
Revenge Motivation	198	2.18	.92	1.00	5.00	.89
Avoidance Motivation	198	3.57	.92	1.00	5.00	.91
Vengeance	198	2.85	.95	1.10	5.20	.94
**CHINESE SAMPLE**						
Revenge Motivation	223	3.00	.87	1.00	5.00	.87
Avoidance Motivation	223	3.63	.70	1.00	5.00	.84
Vengeance	223	3.64	.87	1.55	7.00	.89

Supplementary Table 2. Tests of normality for all variables in the German and the Chinese sample

	Kolmogorov-Smirnov			Shapiro-Wilk		
	**Statistic**	**df**	***p***	**Statistic**	**df**	***p***
**GERMAN SAMPLE**						
Narcissism	.067	198	.031	.994	198	.610
Machiavellianism	.059	198	.095	.990	198	.196
Psychopathy	.079	198	.005	.970	198	<.001
Emotional Manipulation	.095	198	<.001	.959	198	<.001
Revenge Motivation	.133	198	<.001	.932	198	<.001
Avoidance Motivation	.106	198	<.001	.940	198	<.001
Vengeance	.082	198	.003	.974	198	<.001
Eyes Test overall	.129	198	<.001	.911	198	<.001
Eyes Test Caucasian	.112	198	<.001	.944	198	<.001
Eyes Test Asian	.115	198	<.001	.933	198	<.001
**CHINESE SAMPLE**						
Narcissism	.088	223	<.001	.989	223	.074
Machiavellianism	.091	223	<.001	.986	223	.030
Psychopathy	.064	223	.026	.986	223	.028
Emotional Manipulation	.088	223	<.001	.974	223	<.001

Revenge Motivation	.071	223	.008	.985	223	.020
Avoidance Motivation	.110	223	<.001	.962	223	<.001
Vengeance	.091	223	<.001	.975	223	<.001
Eyes Test overall	.156	223	<.001	.865	223	<.001
Eyes Test Caucasian	.128	223	<.001	.938	223	<.001
Eyes Test Asian	.164	223	<.001	.873	223	<.001

Supplementary Table 3. Descriptive statistics for all variables in the German and Chinese sample split by gender.

	N	*M*	*SD*	*Min*	*Max*
**GERMAN FEMALES**					
Narcissism	130	2.59	.54	1.22	3.89
Machiavellianism	130	2.76	.55	1.44	4.33
Psychopathy	130	1.98	.58	1.11	3.44
Emotional Manipulation	130	2.95	.60	1.00	4.60
Revenge Motivation	130	2.06	.87	1.00	5.00
Avoidance Motivation	130	3.51	.91	1.00	5.00
Vengeance	130	2.62	.91	1.10	5.05
Eyes Test overall	130	.66	.10	.25	.85
Eyes Test Caucasian	130	.71	.11	.36	.92
Eyes Test Asian	130	.61	.11	.14	.86
**GERMAN MALES**					
Narcissism	68	2.62	.51	1.11	4.00
Machiavellianism	68	3.30	.58	1.67	4.78
Psychopathy	68	2.32	.47	1.33	3.56
Emotional Manipulation	68	3.18	.51	1.53	4.13
Revenge Motivation	68	2.40	.97	1.00	4.80
Avoidance Motivation	68	3.67	.94	1.14	5.00

Vengeance	68	3.30	.87	1.45	5.20
Eyes Test overall	68	.62	.11	.24	.81
Eyes Test Caucasian	68	.65	.13	.19	.86
Eyes Test Asian	68	.58	.12	.22	.78
**CHINESE FEMALES**					
Narcissism	105	2.90	.41	1.89	3.89
Machiavellianism	105	3.14	.55	1.67	4.89
Psychopathy	105	2.22	.49	1.33	3.22
Emotional Manipulation	105	2.94	.54	1.27	4.73
Revenge Motivation	105	3.11	.80	1.20	5.00
Avoidance Motivation	105	3.79	.58	2.14	5.00
Vengeance	105	3.59	.85	1.70	5.90
Eyes Test overall	105	.67	.10	.25	.82
Eyes Test Caucasian	105	.62	.11	.28	.83
Eyes Test Asian	105	.73	.12	.22	.92
**CHINESE MALES**					
Narcissism	118	2.98	.47	1.78	4.22
Machiavellianism	118	3.37	.60	2.00	5.00
Psychopathy	118	2.46	.57	1.11	3.89
Emotional Manipulation	118	3.15	.62	1.27	5.00

Revenge Motivation	118	2.91	.92	1.00	5.00
Avoidance Motivation	118	3.48	.77	1.00	5.00
Vengeance	118	3.69	.89	1.55	7.00
Eyes Test overall	118	.63	.14	.18	.83
Eyes Test Caucasian	118	.58	.14	.17	.83
Eyes Test Asian	118	.68	.15	.17	.92

Supplementary Table 4. Non-parametric descriptive statistics for all variables in the German and Chinese sample split by gender.

	**Gender x Culture**	***N***	**Mean Rank**
Age	Chinese female	105	119.78
	Chinese male	118	149.83
	German female	130	279.24
	German male	68	327.54
	Total	421	
Narcissism	Chinese female	105	238.19
	Chinese male	118	256.20
	German female	130	166.05
	German male	68	176.51
	Total	421	
Machiavellianism	Chinese female	105	215.10
	Chinese male	118	258.10
	German female	130	142.47
	German male	68	253.96
	Total	421	
Psychopathy	Chinese female	105	210.81
	Chinese male	118	258.95
	German female	130	155.96

	German male	68	233.32
	Total	421	
Emotional Manipulation	Chinese female	105	183.05
	Chinese male	118	230.83
	German female	130	194.63
	German male	68	251.04
	Total	421	
Revenge Motivation	Chinese female	105	272.64
	Chinese male	118	248.39
	German female	130	140.72
	German male	68	185.28
	Total	421	
Avoidance Motivation	Chinese female	105	235.44
	Chinese male	118	187.87
	German female	130	201.39
	German male	68	231.76
	Total	421	
Vengeance	Chinese female	105	246.04
	Chinese male	118	263.36
	German female	130	132.33

	German male	68	216.45
	Total	421	
Eyes Test overall	Chinese female	105	245.49
	Chinese male	118	199.86
	German female	130	216.40
	German male	68	166.76
	Total	421	
Eyes Test Caucasian	Chinese female	105	184.23
	Chinese male	118	155.67
	German female	130	276.56
	German male	68	223.03
	Total	421	
Eyes Test Asian	Chinese female	105	288.35
	Chinese male	118	243.91
	German female	130	157.22
	German male	68	137.26
	Total	421	

Supplementary Table 5. Non-parametric tests on cultural differences for all variables.

	**Mann-Whitney-U**	**Wilcoxon-W**	***Z***	***p***
Sex	17977.000	42953.000	-3.825	<.001
Age	5281.000	30257.000	-13.801	<.001
Narcissism	13888.500	33589.500	-6.588	<.001
Machiavellianism	16089.500	35790.500	-4.813	<.001
Psychopathy	16439.000	36140.000	-4.532	<.001
Emotional Manipulation	21482.000	46458.000	-.478	.633
Revenge Motivation	11192.000	30893.000	-8.751	<.001
Avoidance Motivation	21914.500	46890.500	-.131	.896
Vengeance	12220.000	31921.000	-7.912	<.001
Eyes Test overall	19770.500	39471.500	-1.854	.064
Eyes Test Caucasian	12736.500	37712.500	-7.518	<.001
Eyes Test Asian	10071.500	29772.500	-9.659	<.001
Eyes Test Own Culture	19352.000	39053.000	-2.194	.028
Eyes Test Other Culture	21546.000	41247.000	-.428	.669
Experience with Other Culture	11598.500	31299.500	-8.879	<.001

Supplementary Table 6. Non-parametric tests on gender differences in the German and Chinese sample.

	Mann-Whitney-U	Wilcoxon-W	*Z*	*p*
**GERMAN SAMPLE**				
Age	2890.500	11405.500	-4.022	<.001
Narcissism	4146.500	12661.500	-.716	.474
Machiavellianism	2155.500	10670.500	-5.924	<.001
Psychopathy	2685.500	11200.500	-4.539	<.001
Emotional Manipulation	3209.000	11724.000	-3.167	.002
Revenge Motivation	3488.000	12003.000	-2.442	.015
Avoidance Motivation	3824.500	12339.500	-1.558	.119
Vengeance	2547.000	11062.000	-4.893	<.001
Eyes Test overall	3338.000	5684.000	-2.832	.005
Eyes Test Caucasian	3246.500	5592.500	-3.077	.002
Eyes Test Asian	3868.000	6214.000	-1.449	.147
**CHINESE SAMPLE**				
Age	5023.000	10588.000	-2.652	.008
Narcissism	5545.500	11110.500	-1.355	.175
Machiavellianism	4835.000	10400.000	-2.834	.005
Psychopathy	4646.000	10211.000	-3.227	.001
Emotional Manipulation	4721.000	10286.000	-3.069	.002

Revenge Motivation	5498.500	12519.500	-1.452	.146
Avoidance Motivation	4686.500	11707.500	-3.147	.002
Vengeance	5525.000	11090.000	-1.394	.163
Eyes Test overall	4891.500	11912.500	-2.715	.007
Eyes Test Caucasian	5230.000	12251.000	-2.014	.044
Eyes Test Asian	4800.000	11821.000	-2.912	.004

Supplementary Table 7. Partial rank-correlation coefficients between the DT traits (Spearman's ρ) and all variables under investigation (including Vengefulness), controlled for the respective other two members of the DT and age.

**GERMAN SAMPLE**		**Females** *N* = 130			**Males** *N* = 68	
	**N**	**M**	**P**	**N**	**M**	**P**
Revenge Motivation	.05	.25**	**.54****	-.27*	.13	.30*
Vengeance	.01	**.32****	**.56****	-.30*	.14	**.49****
Avoidance Motivation	-.02	.24**	.10	-.20	.22	-.09
Emotional Manipulation	.20*	**.37****	.12	.21	*.34***	.19
Eyes Test Caucasian	-.10	.09	-.16	.07	-.16	-.22
Eyes Test Asian	-.12	-.04	-.11	-.07	-.18	-.26*
**CHINESE SAMPLE**		**Females** *N* = 105			**Males** *N* = 118	
	**N**	**M**	**P**	**N**	**M**	**P**
Revenge Motivation	.09	.25*	.23*	-.23*	.21*	*.27***
Vengeance	.09	**.38****	**.42****	-.06	.19*	**.38****
Avoidance Motivation	-.09	.08	-.12	-.20*	.10	-.05
Emotional Manipulation	.14	**.38****	.25*	.05	**.36****	.22*
Eyes Test Caucasian	.07	.16	-.20*	-.10	.16	-.22*
Eyes Test Asian	.16	-.13	-.17	.09	.14	-.15

**p<.05; **p<.01, two-tailed. Numbers in bold indicate effect sizes surviving Bonferroni-correction (α≤p/18=.05/18=.0028 for 18 correlations per cultural/gender group). The number in italic represents an effect size marginally failing Bonferroni-correction (p≤.005).*

Supplementary Data 2. Results for Vengefulness

Psychopathy showed positive associations with Vengefulness as measured with the Vengeance Scale and the TRIM-12 Revenge Motivation subscale in all samples. Effect sizes were higher in German females compared to the Chinese female and male sample for Revenge Motivation and Vengeance (both $z>|2.54|$, $p<.05$), but didn't differ significantly from the German male sample on both scales (both $z<|.1.93|$, $p>.05$). Machiavellianism was significantly positively correlated with Revenge Motivation and Vengeance in all subsamples, with exception of German males. Associations were equal in the three remaining samples (all $z<|1.53|$, $p>.05$). Narcissism showed significant negative relations with Revenge Motivation in both male subsamples with same effect sizes ($z=|.28|$, $p=.78$).

In terms of Avoidance Motivation, significant positive associations were found with Machiavellianism, but only in the German female sample. In both male samples, however, Avoidance Motivation was negatively related to Narcissism, with a significant effect in the Chinese group only. Fisher's z-tests indicated no significant difference between the two correlations ($z=0$, $p=1.00$). These effects, however, would not survive Bonferroni correction for multiple testing.
