# Supplementary figures and images for: The Dark Side of Emotion Recognition – Evidence From Cross-Cultural Research in Germany and China
Source: Front Psychol. 2020 Jul 9;11:1132. doi: 10.3389/fpsyg.2020.01132 (PMC7363803; doi:10.3389/fpsyg.2020.01132)

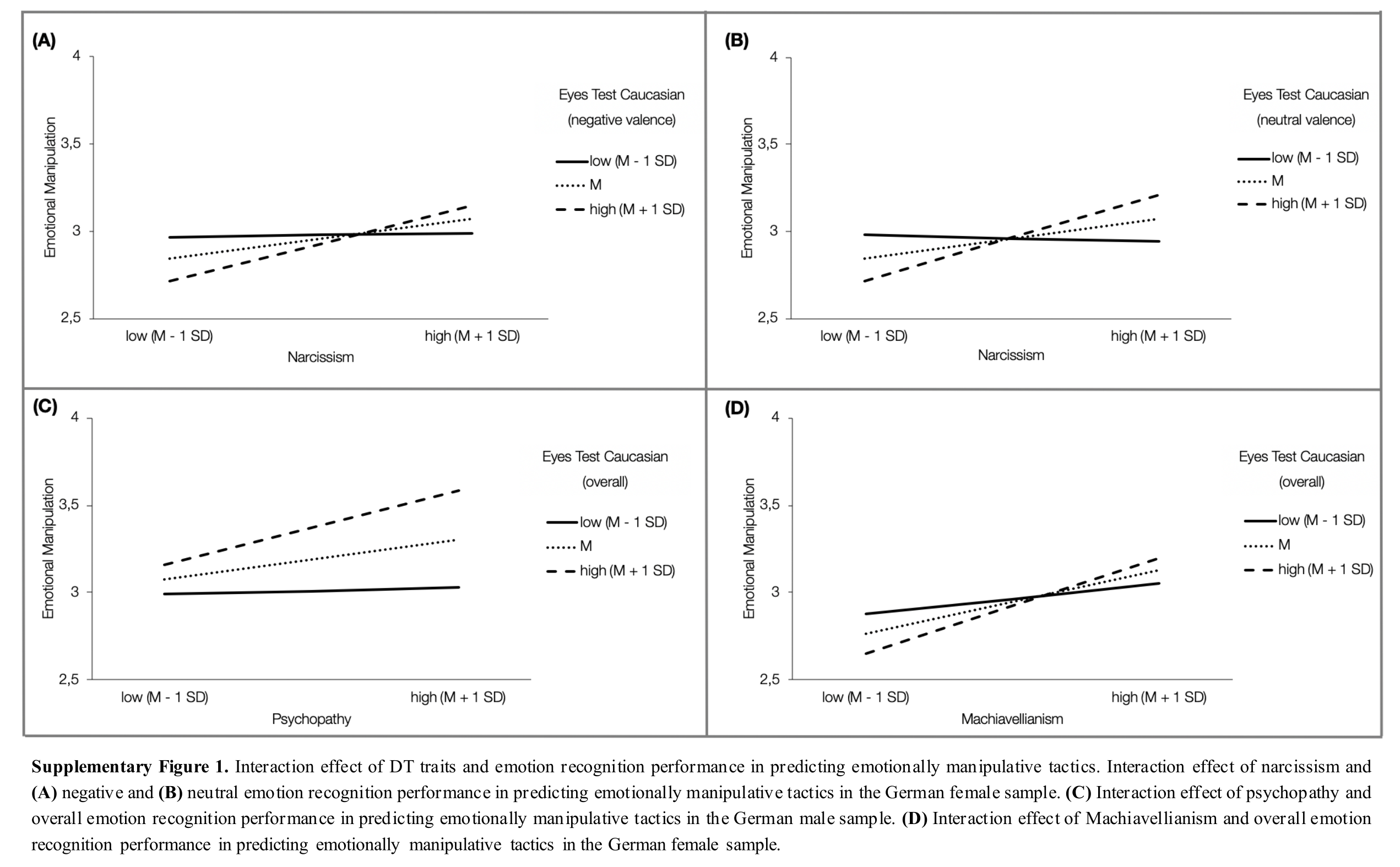

Supplement: Supplementary file 2 [file Image_1.tiff]
